# In-vivo confocal microscopy predicts cytomegalovirus as the cause of chronic or recurrent anterior uveitis among Chinese

**DOI:** 10.1007/s00417-024-06561-3

**Published:** 2024-07-01

**Authors:** Stephanie Hiu-wai Kwok, Ka Wai Kam, Eugenie Mok, Alvin L. Young

**Affiliations:** 1 Department of Ophthalmology & Visual Sciences, Prince of Wales Hospital, Shatin, New Territories Hong Kong SAR; 2 Department of Ophthalmology & Visual Sciences, Alice Ho Miu Ling Nethersole Hospital, Taipo, New Territories Hong Kong SAR; 3 Department of Ophthalmology & Visual Sciences, The Chinese University of Hong Kong, Prince of Wales Hospital, Shatin, New Territories Hong Kong SAR

**Keywords:** Cornea, Uveitis, Cytomegalovirus, Confocal microscopy

## Abstract

**Purpose:**

To evaluate and compare endothelial features by in-vivo confocal microscopy (IVCM) in Chinese eyes with chronic or recurrent anterior uveitis (AU) with and without cytomegalovirus (CMV).

**Methods:**

A double-masked, cross-sectional case-control study at a tertiary eye clinic.

**Results:**

Thirty eyes of 30 subjects were analyzed. Fifteen eyes (50%) were CMV positive, while fifteen eyes were negative for herpes simplex virus, varicella zoster virus and CMV. Absence of pseudoguttata was the strongest, independent risk factor for CMV (OR 34.53, 95% CI: 1.84-648.02, *p* = 0.018), followed by severe iris depigmentation (OR 31.45, 1.02-965.81, *p* = 0.048) and low corneal endothelial cell density (ECD) (OR 14.79, 1.14–191.30, *p* = 0.039) on univariable regression. All three remained statistically significant after adjustment. The combination of absence of pseudoguttata and low ECD on IVCM achieved a similar predictive value as iris depigmentation examination.

**Conclusion:**

Absence of pseudoguttata on IVCM was an independent predictor of positive CMV detection after adjusting for iris depigmentation and corneal endothelial cell density. The addition of this feature to severe iris depigmentation and low corneal ECD can increase the positive predictive value of detecting CMV. IVCM was a useful non-invasive tool to predict CMV in patients with chronic or recurrent AU.

## Key message

***What is known:***

• Early detection of CMV can guide use of CMV-specific treatment for better clinical outcomes.

• The gold standard of polymerase chain reaction (PCR) detection of viral DNA in aqueous fluid involves an invasive procedure with known risk of false negative results.

***What is new:***

• Absence of pseudoguttata on the non-invasive in-vivo confocal microscopy (IVCM) is an independent risk factor for CMV detection in aqueous fluid.

• Performing IVCM in patients with chronic or recurrent anterior uveitis can increase the positive predictive value of detecting CMV via aqueous tapping, hence stratifying patients with a higher risk of CMV involvement to receive a more invasive investigative procedure.

## Introduction

Cytomegalovirus (CMV) is increasingly recognized as a cause of anterior uveitis (AU) in immunocompetent individuals, especially in Southeast Asia. Unlike immunocompromised hosts, CMV targets the anterior segment in apparently healthy subjects causing repeated bouts or persistent inflammation. If the infection is left untreated, the condition can lead to sight-threatening complications, such as cataract, corneal decompensation and glaucoma [1]. Even with appropriate antiviral treatment, eyes with CMV are at a higher risk of post-operative complications such as uveitic recurrence and graft failure following intraocular surgery, or corneal transplantation [2]. While a positive polymerase chain reaction (PCR) to CMV DNA is commonly used as the gold standard in diagnosing CMV AU, negative results can still occur. This may be due to a small volume of aqueous aspirate, sensitivity of the PCR, stage of infection, and false negatives, which can be frustrating for both clinicians and patients. To address this issue, it is crucial to consider other alternative supportive evidence to differentiate eyes with negative PCR results. Moreover, anterior chamber (AC) tapping is an invasive procedure that carries inherent risks, such as infection, wound leak, and inadvertent lens or corneal injury.

In our experience, we observed that even among Asians, clinical features of CMV AU could vary between ethnicities, which is in line with recent literature comparing Asian and Caucasian eyes with CMV AU [3]. For instance, coin-shaped keratic precipitates (KP), which was reported in up to 70.6% of Japanese patients, [4] was only seen in less than 20% of our Chinese patients [5]. Endotheliitis was much less common in our population compared to Japan. On the other hand, iris depigmentation was a prominent feature in our locality [6]. Nonetheless, there was no specific clinical feature that was linked to an individual ethnicity, [7] and these findings were dependent on a timely, and meticulous slit-lamp examination. Some of these signs observed in CMV AU are also observed in other viral uveitis caused by herpes simplex virus (HSV), rubella virus (RV) and varicella zoster virus (VZV). Given the predilection of CMV for corneal endothelium, we were interested in the endothelial features of eyes with a positive CMV PCR, and whether in-vivo confocal microscopy (IVCM) plays a role in predicting a diagnosis of CMV AU [8]. 

Previous studies have reported morphological changes in corneal endotheliitis due to HSV, VZV, CMV and Epstein-Barr virus (EBV) [9]. In particular, studies on CMV endotheliitis have focused on describing owl’s eyes morphology, which is a hallmark for CMV viral inclusion bodies [10]. However, no studies to date have used IVCM features to predict a diagnosis of CMV AU. We aim to investigate the utility of IVCM as an auxiliary tool in differentiating CMV from idiopathic AU.

## Methods

We conducted a double-masked, cross-sectional case-control study on subjects recruited from our published, prospective cohort of Chinese adults with chronic or recurrent AU [6]. Eyes were divided into two groups based on the aqueous PCR result after subject recruitment and baseline slit-lamp examination. Group 1 had positive CMV and negative HSV and VZV PCR, whereas group 2 had negative PCR for all three viruses. In this post-hoc study, we invited all subjects for a slit lamp biomicroscopy and IVCM by a single investigator (S.H.K), who was masked to the CMV status of the subject at the time of examination. A standard imaging protocol was adopted [11]. Representative and analyzable endothelial images were reviewed and graded by a second senior investigator (K.W.K.) who was also masked to group allocation. Clinical and imaging findings were then compared between the two groups. The study adhered to the tenets of the Declaration of Helsinki and was approved by the Joint Chinese University of Hong Kong New Territories East Cluster Clinical Research Ethics Committee (2020.423). All subjects provided written informed consent before participation.

### Inclusion and exclusion criteria

Subjects who were recruited in our earlier prospective cohort were eligible for this study. The inclusion and exclusion criteria were published previously [6]. Consecutive adult subjects aged 18 or older, who were diagnosed with recurrent or chronic anterior uveitis, with or without ocular hypertension, were eligible for this study. The definition of chronic or recurrent AU was as per the Standardization of Uveitis Nomenclature classification. Subjects with active corneal infections other than CMV, significant corneal scarring, history of ocular trauma, infectious keratitis, refractive surgeries or keratoplasty, immunodeficiency, active immunocompromised state, active malignancy, or uveitis with a known systemic association were excluded.

### Sample size calculation

The sample size was calculated with StataCorp Version 13.0 and based on Choi’s study [12] which identified a difference of 736 cells/mm^2^ in endothelial cell density between eyes with CMV-positive and CMV-negative chronic or recurrent, hypertensive AU. The minimum sample size required to achieve 90% power and 5% significance level is 11 subjects per arm. Assuming a dropout rate of 20%, we aimed to recruit a total of 28 subjects, with at least 14 subjects in each group.

### Study procedure

All subjects were recruited and examined by the principal investigator (S.H.K.). Demographic data were collected using a standardized datasheet. The presence of glaucoma was defined as a regular use of anti-glaucomatous medication at the time of assessment for presence of structural and/or functional deficit on optical coherence tomography or automated perimetry, which excluded temporaneous prescription for intraocular pressure spike during a hypertensive flare.

Slit lamp biomicroscopy was performed to assess for signs of inflammation including corneal clarity, AC activity, presence of KP and/or posterior synechiae. Iris depigmentation was categorized into none, mild, moderate, or severe. Details of the assessment method for iris depigmentation have been published [6]. In short, iris depigmentation was classified as mild, moderate or severe when 25% or less, 50% or less, or more than 50% of any quadrant of iris showed a loss of brown pigments.

IVCM (Heidelberg Retina Tomograph III Rostock Corneal Module) (HRTIII) was performed at the corneal apex, as well as areas with KP located on slit lamp biomicroscopy. Three best images (i.e. well-focused with good contrast) taken at the level of the endothelium were selected for analysis. These images were evaluated by a single masked experienced grader (K.W.K.), and were graded against reported endothelial features of viral AU, which included owl’s eye morphology, anomalous nucleus, guttata, pseudoguttata, enlarged intercellular gaps, spot-like holes, loss of defined cell boundaries, endothelial denudation, and infiltration of inflammatory cells into the endothelial layer [9, 13]. Endothelial cell density (ECD) was calculated using the manual cell-count processing mode within the analytical software provided with the HRTIII. Cells that were partially within the area analyzed were only counted along the right and lower margins.

### Statistical analyses

In order to avoid non-independence of eyes, one eye of patients with bilateral disease was randomly excluded (www.randomizer.org) before analysis. The results were analyzed using SPSS software version 26 (SPSS Inc., Chicago, IL). Demographics, past ocular and medical history, and biomicroscopic and IVCM findings were compared between subjects with and without CMV using independent t or Mann Whitney U test for continuous data, and chi-square test or Fisher’s exact test for categorical data as appropriate.

Associations between demographic, clinical and confocal features, and CMV status were calculated by univariate and multiple logistic regressions. The median value of endothelial cell density was used to binarize our study population so that the data could be analysed as both continuous and categorical variables. A *p* < 0.05 was considered statistically significant. Positive and negative predictive values (PPV and NPV) were calculated by individual and also combinations of demographic, clinical and confocal features that achieved statistical significance in the regression models.

## Results

### Demographics

Thirty eyes from 30 subjects were recruited and analyzed. The mean age of subjects was 60.99 ± 7.66 years old. There was a male predominance (M: F = 2). Fifteen eyes (50%) were positive for CMV DNA, while all eyes were negative for HSV and VZV DNA. Eyes with or without CMV had comparable disease duration, hypertensive characteristics, and past ocular history, except for a doubled incidence of glaucoma in the CMV-positive group (*p* = 0.025). The two groups were similar in terms of slit-lamp findings. AC cells were absent in all eyes, while KP was observed in more than two-thirds of subjects in both groups. Eyes with CMV had significantly greater amount, as well as severity of iris depigmentation than eyes without CMV (*p* = 0.04, and *p* = 0.009, respectively) (see Table 1).

**Table 1:** Demographics, past ocular and medical history, clinical and IVCM features of the 30 subjects

	CMV positive (*n* = 15)	%	CMV negative (*n* = 15)	%	*P* value
**Demographic features**					
Age (years)	61.65 ± 6.85	N/A	60.32 ± 8.58	N/A	0.643
Male sex	12	80%	8	53.3%	0.121
**Past ocular and medical history**					
Duration of AU (months)	98.27 ± 60.995	N/A	116.20 ± 59.622	N/A	0.422
Hypertensive AU	15	100%	13	86.7%	0.241
History of cataract surgery	10	66.7%	6	40%	0.143
History of any ocular surgery	11	73.3%	6	40%	0.065
Glaucoma	12	80%	6	40%	**0.025**
Diabetes	4	26.7%	5	33.3%	0.500
**Clinical features on day of IVCM**					
Any steroid use	8	53.3%	10	66.7%	0.456
AC cells grade 1 or above	0	0%	0	0%	1
Any KPs	11	73.3%	10	66.7%	0.500
PS	1	6.7%	1	6.7%	0.759
Immune ring	0	0%	1	6.7%	0.500
Corneal edema	0	0%	0	0%	1
Any iris depigmentation	14	93.3%	9	60%	**0.040**
Iris depigmentation quadrants					
1 quadrant	0	0%	0	0%	0.189
2 quadrants	3	20%	2	13.3%	
3 quadrants	1	6.7%	1	6.7%	
4 quadrants	10	66.7%	6	40%	
Iris depigmentation severity					
Mild	3	20%	7	46.7%	**0.009**
Moderate	4	26.7%	1	6.7%	
Severe	7	46.7%	1	6.7%	
**IVCM features**					
Endothelial cell density	1729.07 ± 512.536	N/A	2163.07 ± 566.691	N/A	**0.036**
Owl’s eyes	3	20%	4	26.7%	0.500
Anomalous nuclei	12	80%	12	80%	1
Dark nuclei	9	60%	9	60%	1
Light nuclei	2	13.3%	3	20%	0.500
Bi/poly nuclei	7	46.7%	4	26.7%	0.256
Guttata	0	0%	0	0%	N/A
Pseudoguttata	2	13.3%	8	53.3%	**0.020**
Small black dots at cellular borders	15	100%	15	100%	N/A
Broadened cellular borders	8	53.3%	10	66.7%	0.456
Loss of defined cell boundaries	10	66.7%	8	53.3%	0.456
Spot-like holes	2	13.3%	1	6.7%	0.500
Endothelial denudation	3	20%	0	0%	0.112
Infiltration of inflammatory cells	1	6.7%	2	13.3%	0.500

IVCM: in vivo confocal microscopy; CMV: cytomegalovirus; AU: anterior uveitis; AC: anterior chamber; KP: keratic precipitates; PS: posterior synechiae

### IVCM features

**Fig. 1:**
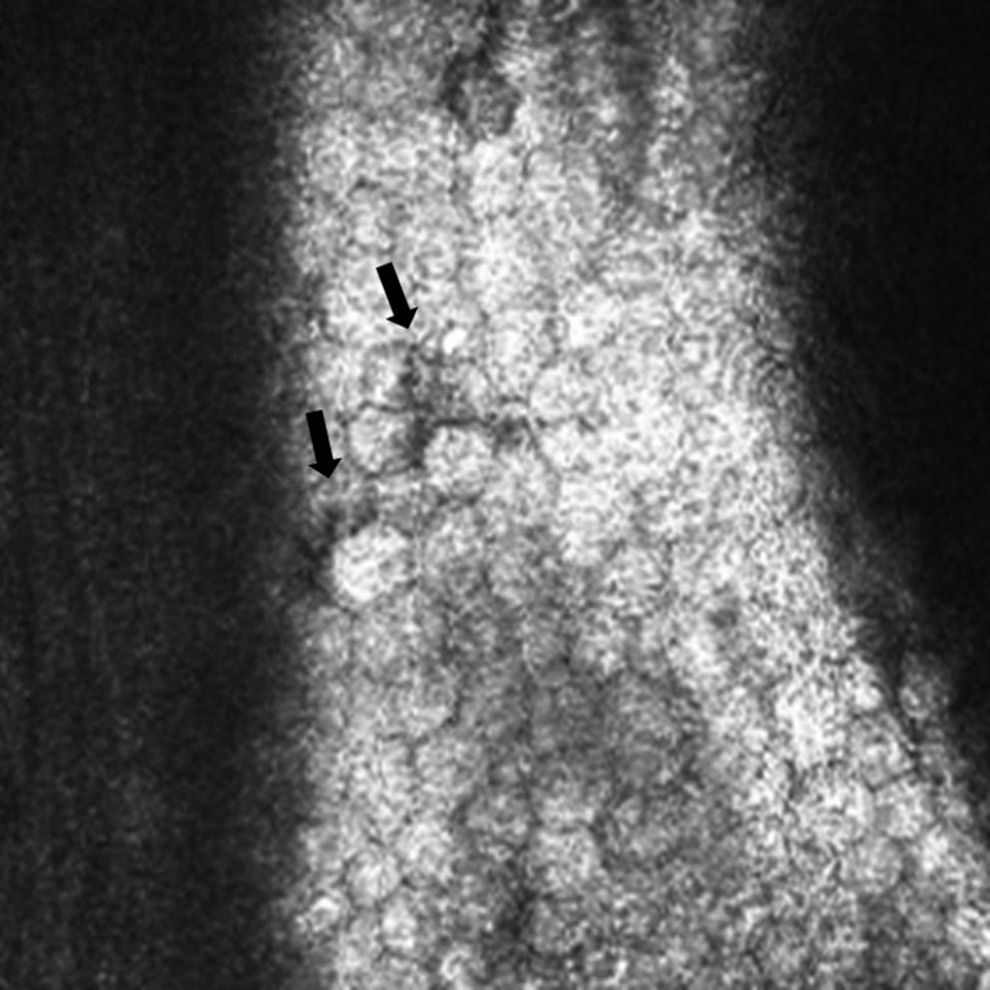
Pseudoguttata identified on in-vivo confocal microscopy

The median ECD of our patient cohort was 1892.5 cells/mm^2^. Eyes with CMV had a significantly lower ECD than eyes without CMV (*p* = 0.036). Pseudoguttata (Fig. 1) was detected more frequently in eyes without CMV (*p* = 0.020), whilst the detection of other endothelial features was comparable. Only three eyes (20%) with CMV had owl’s eye cells, whilst four eyes (26.7%) with pan-negative PCR were judged to have owl’s eye.

### Risk factors

Table 2 summarizes the results of univariate logistic regression which revealed severity of iris depigmentation as a significant risk factor for CMV as it was associated with a greater odds of CMV (OR 4.05, 95%CI 1.53–10.73, *p* = 0.005). The presence of pseudoguttata was on the other hand a protective factor, which was associated with a lower odds of CMV. (OR 0.135, 95% CI 0.02–0.82, *p* = 0.029). A greater endothelial cell density on IVCM might be a protective factor against CMV but the statistical significance was only marginal (*p* = 0.05).

**Table 2:** Associations of demographic, clinical and IVCM features in predicting positive CMV tap

	Odds ratio	95% CI	*P* value
**Univariate analysis**			
**Demographic features**			
Age	1.024	0.930–1.127	0.631
Sex	3.500	0.692–17.714	0.130
Diabetes	0.727	0.151–3.493	0.691
**Clinical features**			
Any KPs	1.375	0.286–6.603	0.691
Coin-shaped KPs	0.196	0.019–2.017	0.171
Linear KPs	N/A		
PS	N/A		
Immune ring	N/A		
Corneal edema	N/A		
Any iris depigmentation	9.333	0.958–90.940	0.054
Iris depigmentation quadrants	1.634	0.986–2.708	0.057
Iris depigmentation severity	4.047	1.526–10.733	**0.005**
**Confocal features**			
Endothelial cell density	0.998	0.997-1.000	**0.050**
Owl’s eyes	0.688	0.125–3.786	0.667
Anomalous nuclei	N/A		
Dark nuclei	N/A		
Light nuclei	0.615	0.087–4.341	0.626
Bi/poly nuclei	2.406	0.521–11.104	0.260
Guttata	N/A		
Pseudoguttata	0.135	0.022–0.816	**0.029**
Small black dots at cellular borders	N/A		
Broadened cellular borders	0.571	0.130–2.503	0.458
Loss of defined cell boundaries	1.750	0.400-7.664	0.458
Spot-like holes	2.154	0.174–26.672	0.550
Endothelial denudation	N/A		
Infiltration of inflammatory cells	0.464	0.037–5.749	0.550
**Multivariate analysis**			
Severe iris depigmentation	31.446	1.024–965.810	**0.048**
Low ECD below median^1^	14.790	1.143-191.301	**0.039**
NO pseudoguttata	34.531	1.840-648.022	**0.018**

IVCM: in vivo confocal microscopy; CMV: cytomegalovirus; KP: keratic precipitates; PS: posterior synechiae

1: Median ECD of our patient cohort was 1892.5 cells/mm^2^

In multivariable logistic regression, severe iris depigmentation, reduced corneal ECD, and absence of pseudoguttata on IVCM were independently associated with higher risks of CMV. (See Table 2). Among the three, the absence of pseudoguttata on IVCM was the strongest determinant for CMV, regardless of the severity of iris depigmentation and endothelial cell density (adjusted OR = 34.53, 95% CI 1.84–648.02, *p* = 0.018).

### Positive and negative predictive values

Severe iris depigmentation, reduced corneal ECD below median and absence of pseudoguttata on IVCM were evaluated for their effect on the sensitivity, specificity, PPV and NPV of CMV DNA in aqueous humor. (See Table 3) Absence of pseudoguttata on IVCM was the most sensitive (86.7%) for identifying eyes with CMV, followed by reduced corneal ECD on IVCM (60.0%) and severe iris depigmentation (46.7%). On the other hand, severe iris depigmentation had the greatest positive predictive value for CMV (87.5%) and was the most specific sign for CMV (93.3%). This was followed by reduced corneal ECD (66.7%) and absence of pseudoguttata on IVCM (53.3%).

**Table 3:** Sensitivity, specificity, positive and negative predictive values using different combinations of clinical and IVCM variables

	CMV positive	CMV negative	Sensitivity	Specificity	PPV	NPV
**One variable**						
Severe ID	7 (46.7%)	1 (6.7%)	46.7%	93.3%	87.5%	63.6%
Low ECD below median^1^	10 (66.7%)	5 (33.3%)	60%	66.7%	60%	60%
No pseudoguttata	13 (86.7%)	7 (46.7%)	86.7%	53.3%	65%	80%
**Two variables**						
Severe ID + low ECD	5 (33.3%)	0	33.3%	100%	100%	60%
Severe ID + No pseudoguttata	6 (40%)	0	40%	100%	100%	62.5%
Low ECD + No pseudoguttata	8 (53.3%)	1 (6.7%)	53.3%	93.3%	88.9%	66.7%
**Three variables**						
Severe ID + low ECD + No pseudoguttata	4 (26.7%)	0	26.7%	100%	100%	57.7%

IVCM: in vivo confocal microscopy; CMV: cytomegalovirus; ID: iris depigmentation; ECD: endothelial cell density

1: Median ECD of our patient cohort was 1892.5 cells/mm^2^

When any of the two variables were combined, there was an increase in the PPV for CMV. Among the combinations, reduced corneal ECD and absence of pseudoguttata had the best overall profile with the greatest sensitivity, and reasonably high specificity, PPV and NPV. On the other hand, utilizing all three variables reduced the sensitivity and NPV.

## Discussion

For the first time, we conducted a case-control study to investigate the associations of slit-lamp and confocal features and the CMV status in Chinese eyes with chronic or recurrent AU. Our data showed that classical features such as owl’s eye cells could be observed in both eyes with positive and negative CMV PCR. This supports a possible false negative PCR result and exemplifies the limitation of aqueous PCR. In contrast, eyes with absence of pseudoguttata and lower corneal ECD, and more severe iris depigmentation were independently associated with a higher predictive risk of CMV, either alone or in combination. In particular, the combination of absence of pseudoguttata and severe iris depigmentation raised the PPV to 100% and NPV to 62.5%. The combination of absent pseudoguttata and low corneal ECD, both identified on IVCM, also achieved a comparable PPV of 100% and NPV of 60%. Although we had shown that in carefully selected eyes, iris depigmentation could be a potential biomarker for CMV AU, it was imperative to consider alternative causes for pigment loss in iris, such as prior intraocular surgery, iatrogenic iris injury, acutely elevated intraocular pressure (for example, acute angle closure), pseudoexfoliation, or underlying genetic predisposition. The merit of utilizing IVCM in predicting CMV was the independence from a time-sensitive slit-lamp examination, and the subjectivity in grading iris depigmentation.

In our cohort, identification of pseudoguttata was more frequent in eyes without CMV. Pseudoguttata was first described by Krachmer and coworkers in 1981, referring to hyporeflective elevated spots seen among regularly arranged endothelial cells in eyes with corneal inflammation, that disappear when inflammation resolves [14]. Scanning and transmission electron microscopy of these lesions suggested transient endothelial cell edema to be the culprit, which could be secondary to infection. Histologically, unlike true guttata, pseudoguttata lacks excrescences around the endothelial cells. Owing to its transient nature, it is a scarcely reported clinical feature, and scarce reports on histology. The largest series of pseudoguttata reported its occurrence in 44 eyes suffering from contact lens-related keratitis, keratoconjunctivitis, corneal epithelial defect, corneal foreign body and keratitis [15]. In comparison, true guttata appears as hyporeflective elevated spots with a hyperreflective white dot in the center, and forms due to focal thickening of Descemet’s membrane [14, 15]. These lesions do not resolve with resolution of inflammation.

Other than the pseudoguttata, there was no other significant difference in confocal signs of endotheliitis between the two groups. Interestingly, while none of our patients had detectable anterior chamber cells at the time of assessment, pseudoguttata was identified in 13.3% of CMV positive patients and 53.3% of CMV negative patients. This was in contrast to a previous report which described pseudoguttata to resolve when the inciting episode of anterior segment inflammation subsided [15]. This could be due to a difference in the disease duration, or the stage of infection between their study and ours. It is unclear how long pseudoguttata would remain present after clinically detectable anterior chamber inflammation resolves. Another possible explanation was that IVCM performed more superiorly than slit lamp examination alone in detecting subclinical inflammation in the corneal endothelium, hence the results reflecting a higher proportion of subclinical inflammation present in our CMV negative patients.

Our current cohort replicated results from our earlier study, showing that severe or diffuse iris depigmentation, even when assessed at a much later timepoint after AC tapping, could still effectively differentiate CMV AU from eyes with pan-negative PCR [6]. Nonetheless, there was always an element of subjectiveness and operator-dependence in identifying and quantifying iris depigmentation, despite a standard description. Hence, we hope that IVCM could provide a more objective assessment, that was less sensitive to time. The combination of at least one confocal feature with either iris depigmentation or absence of pseudoguttata was shown to improve the predictive accuracy for CMV.

The majority of eyes in our cohort experienced hypertensive episodes during flares. However, there was a significantly larger proportion of eyes in the CMV positive group suffering from glaucoma (80%, vs. 40% in CMV negative group). This was in line with the findings by Shirahama and coworkers, who identified significantly higher prevalence and faster progression of secondary glaucoma in patients with CMV AU, when compared to HSV AU and VZV AU [16]. This reiterates the importance of earlier identification of CMV in eyes with AU, for more aggressive monitoring and management of glaucoma.

Our analysis of IVCM and clinical features of CMV AU carries important clinical implications. We showed that a combination of any two of the following factors, absence of pseudoguttata on IVCM, low corneal ECD and severe iris depigmentation effectively increased the prediction of CMV as the cause of AU. Apart from documenting the severity of iris depigmentation during clinical exam, which was subjective and multifactorial in nature, clinicians may utilize IVCM to evaluate for pseudoguttata and corneal ECD in the central cornea for patients with recurrent or chronic AU before AC tapping. IVCM is a non-invasive procedure which poses minimal risk to patients, and provides important data so that clinicians can selectively offer AC tapping to patients with at least two of the following three features: severe iris depigmentation, absence of pseudoguttata on IVCM and low corneal ECD.

Nevertheless, our study had several limitations. First, our patients had a relatively long duration of AU. IVCM was only performed once but at a variable time point after an AC tap. These subjects had received at least 3 months of treatment (topical corticosteroid with or without antiviral) before the start of this study. As stated in the results, all eyes had zero inflammatory cells and clear cornea on the day of IVCM. A prospective study which recruits fresh eyes before AC tapping and offers IVCM on the day of uveitic recurrence might reveal more information especially on any acute endothelial differences, however the presence of severe corneal oedema may affect the image quality of the endothelium during IVCM, or impede localization of keratic precipitates, if any, thus making analyses difficult. The impaired vision during recurrence may also reduce ability to fixate during image capture. Alternatively, a uniform, pre-determined time-point for IVCM may theoretically reduce bias but we could not entirely control for the variable disease course following AC tap and response to treatment. Second, our study was limited by a small sample size, which was reflected in the wide confidence intervals and the predictive values analyses. Nonetheless, the directions of effect were uniform and a study of larger sample size may help refine the precision of data in addition to a proof of concept. Moreover, both the identification of pseudoguttata on IVCM and assessment of iris depigmentation are subject to the experience of the observer. We attempted to mitigate this bias by using one single operator on IVCM with a standard protocol, and another independent, masked observer in grading the IVCM images.

In Chinese eyes with chronic or recurrent CMV AU, it is prudent to note for the extent and severity of iris depigmentation on slit lamp exam, and to monitor for development of glaucoma. Further imaging such as the non-invasive IVCM plays a role in eyes with high clinical suspicion for CMV. Detection of low ECD and absence of pseudoguttata increases the PPV for detection of CMV, which are both important factors to consider when counselling patients for AC tapping in hypertensive, acute or current AU.
